# Colorimetric Detection of *Ehrlichia Canis* via Nucleic Acid Hybridization in Gold Nano-Colloids

**DOI:** 10.3390/s140814472

**Published:** 2014-08-08

**Authors:** Ajima Muangchuen, Piyasak Chaumpluk, Annop Suriyasomboon, Sanong Ekgasit

**Affiliations:** 1 Laboratory of Plant Transgenic Technology and Biosensor, Department of Botany, Faculty of Science, Chulalongkorn University, Bangkok 10330, Thailand; E-Mail: ajimam@nu.ac.th; 2 Department of Animal Husbandry, Faculty of Veterinary Science, Chulalongkorn University, Bangkok 10330, Thailand; E-Mail: annop.suriyasomboon@gmail.com; 3 Sensor Research Unit, Department of Chemistry, Faculty of Science, Chulalongkorn University, Bangkok 10330, Thailand; E-Mail: sanong.e@chula.ac.th

**Keywords:** LAMP, *Ehrlichia canis*, gold nanoparticles

## Abstract

Canine monocytic ehrlichiosis (CME) is a major thick-bone disease of dog caused by *Ehrlichia canis*. Detection of this causal agent outside the laboratory using conventional methods is not effective enough. Thus an assay for *E. canis* detection based on the *p30* outer membrane protein gene was developed. It was based on the *p30* gene amplification using loop-mediated isothermal DNA amplification (LAMP). The primer set specific to six areas within the target gene were designed and tested for their sensitivity and specificity. Detection of DNA signals was based on modulation of gold nanoparticles' surface properties and performing DNA/DNA hybridization using an oligonucleotide probe. Presence of target DNA affected the gold colloid nanoparticles in terms of particle aggregation with a plasmonic color change of the gold colloids from ruby red to purple, visible by the naked eye. All the assay steps were completed within 90 min including DNA extraction without relying on standard laboratory facilities. This method was very specific to target bacteria. Its sensitivity with probe hybridization was sufficient to detect 50 copies of target DNA. This method should provide an alternative choice for point of care control and management of the disease.

## 1. Introduction

Canine monocytic ehrlichiosis (CME), a tropical canine pancytopenia, is a common tick-borne disease caused by *Ehrlichia canis*, a Gram negative obligate parasite that invades and replicates in dog monocytes and macrophages [1]. Common symptoms in dogs include high fever, depression, lethargy, anorexia, lymphadenomegaly, splenomegaly and death [2].

Diagnosis of canine ehrlichiosis is normally based either on hematologic, biochemical, or serologic assays. Among them, serologic techniques such as immunofluorescent antibody (IFA) tests or even standard ELISA [3–5] are routinely employed. Although these assays are well accepted, they relied heavily on laboratory facilities and have some limits in both sensitivities and specificities [6]. Therefore, a rapid, simple and inexpensive detection assay development for *E. canis* is important and is still needed to ensure early treatment with a good prognosis, suitable for early epidemiologic control and measurement.

A PCR assay has been applied and accepted as an alternative solution to detect *E. canis* due to its high sensitivity of checking the incidence in peripheral blood, even at low bacteria levels. Several PCR platforms, targeting a variety of determinants *i.e.*, *16S* rRNA, *p28*, *p30*, *dsb*, *Vir*B9, were developed and demonstrated [7–12]. Although these PCR based techniques were useful for experimental investigations, the limitation of PCR in term of the lack of assay simplicity, its time consumption and its dependence on laboratory facilities both for a thermal cycler device and technical hands-on experience, have created a barrier to field site application of the technique as a point-of-care test approach.

Recently, an alternative method of nucleic acid amplification called Loop-mediated isothermal amplification (LAMP) has been introduced [13]. It provides better specificity, simplicity, and speed in nucleic acid amplification [14]. In the past, our group had previously reported a simple and rapid *E. coli* and gene-related freshness detection based on DNA amplification via LAMP and fluorescence detection via a minor groove DNA binder [15,16], however, nucleic acid detection by LAMP using DNA binder alone was not a sequence specific test. Further steps of confirmation of the DNA sequence in target products either by DNA sequencing or nucleic acid hybridization was still required.

Mirkin *et al.* [17] had reported the first colorimetric detection via the demonstration of thiol-modified single strand DNA as a probe for functionalization on gold nanoparticles, and used them for nucleic acid hybridization. The cross-linking network between probe and DNA induced the aggregation of the gold nanoparticles causing a shift in the surface plasmon resonance from ruby red to purple at 574 nm. However, the use of thiol-modified single strand DNA functionalization of DNA on gold nanoparticles was carried out with difficulty and had a problem with stability [18].

Kanjanawarut and Su [19] pioneered the use of peptide nucleic acid binding to ion-coated gold nanoparticles for modulation of particles' surface properties and performing DNA hybridization. To apply this principle in combination with the plasmonic properties of noble metal nanoparticles for agricultural applications, several demonstrations had been carried out by our group [16]. In one model, an attempt to apply a synthetic peptide nucleic acid probe for target DNA hybridization using non-functionalized gold nanoparticles was conducted for fruit maturity assay in a non-destructive fashion [16]. Presence of target DNA, a negative marker for fruit maturation, affected gold colloid nanoparticles in term of non-aggregation with no color change (still ruby red). However, only in the mature fruit, the expression of a target sucrose transporter marker, *AcSUT1*, was terminated resulting in no target cDNA and no DNA amplification. When this was tested with gold colloid nanoparticles, it induced aggregation of particles which in turn resulted in a plasmonic change of the colloid solution from ruby red to purple, visible by the naked eye. However, the use of a peptide nucleic acid probe was rather restricted due to its technical limitations during synthesis [20,21], its cost effectiveness, and it is still not in popular use.

Since colorimetric detection of DNA using nanoparticles was under focused and most of the reports were theoretical demonstrations based on synthetic DNA, thus in this paper, we had developed an assay for *E. canis* detection using an oligonucleotide as a probe and applied it for real sample tests. This assay was based on the combination of DNA amplification technique called Loop-mediated isothermal amplification (LAMP) and the detection of DNA signals using plasmonic change of gold nanoparticles (AuNPs) upon hybridization with a normal oligonucleotide probe. The use of LAMP enables the tests in real specimens to be conducted rapidly with less reliance on laboratory facilities while signal detection via an oligonucleotide probe allows simple nucleic acid hybridization to occur in gold nanocolloid solution opening the feasibility to perform field site monitoring tests.

## 2. Experimental Section

### 2.1. Bacterial Specimens and DNA Preparation

Bacteria used in this study—*Ehrlichia canis*, *Ehrlichia chaffeensis*, *Anaplasma phagocytophilla*, *Babesia canis*, *Hepatozoon canis* and *Escherichia coli*—were from Faculty of Veterinary Science, Chulalongkorn University. DNA from each specimen was extracted using the Invisorb Spin Blood Mini Kit^TM^ as described in the manufacturer's protocol (Stratec Molecular, Birkenfeld, Germany). For negative control of non-target DNA, LAMP products from *Listeria monocytogenes hly* gene was used. Clinical DNA specemens were from Faculty of Veterinary Science, Chulalongkorn University. *E. canis* positive and negative specimens were screened first using ELISA test kit (SNAP^®^ 4Dx^®^ Plus Test; Idexx laboratory, Westbrook, ME, USA) and the results confirmed using hematogic assay [22] and PCR [8].

### 2.2. Design LAMP Primers and Probe for LAMP Assay

**Figure 1.**
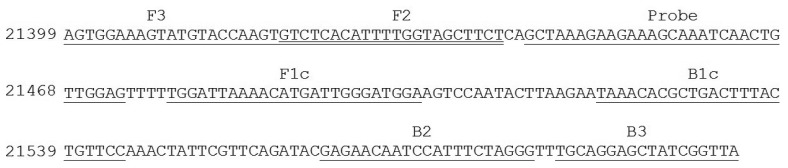
Position of primer and probe on p30 gene, Accession No. AF078553.

Nucleotide sequence of *E. canis p30* gene from GenBank were analyzed and used for the design of the candidate primers. In brief, the corresponding sequences were arranged in FASTA format and aligned using Clastal W program with reference to sequence accession No. AF078553 [23]. The primer set for the targeted gene amplification via LAMP was then designed based on the Primer Explorer program version 4 (Fujitsu System Solution Ltd., Tokyo, Japan). Selected gene primers were scored based on sequence parameter including Tm, E value, and nucleotide sequence similarities. The specificity and melting temperature of the primer set was checked using Basic Local Alignment Search Tool (BLASTn) [24] and Oligocalculator [25], respectively. For a specific oligonucleotide probe design, nucleotide sequence element of *p30* gene between F3 and B3 primer was selected, checked for its specificity using BLASTn program [24] and sent to a custom DNA synthesis service (1st BASE, Singapore). Detail of primer set and probe used were described in Table 1 and Figure 1.

### 2.3. DNA Amplification by LAMP

Each 25 μL of LAMP reaction contained a mixture of 1 μL extracted DNA, 40 pmol of each FIP and BIP primer, 5 pmol of each F3 and B3 primer, 5 mM of each dNTP, 3 mM of MgSO_4_, ThermoPol^TM^ reaction buffer (20 mM Tris-HCl, 10 mM KCl, 10 mM (NH_4_)_2_SO_4_, 2 mM MgSO_4_, 0.1% Triton X-100, pH 8.8) Betaine 1.6 M and 8 U of *Bst* DNA polymerase large fragment (New England Biolabs, Herth, UK). The mixture was incubated at 63 °C for 40 min. No post heat incubation at 80 °C was applied. For determination of DNA products, DNAs from the amplified specimens were separated on 2% agarose TAE gel electrophoresis, and visualized the DNA via UV illumination as described [26].

### 2.4. DNA Detection

Gold nanoparticles 20 nm in size in water solution were prepared and selected under increasing alkaline condition using soluble starch as reducing agent [27]. Probe–gold nanoparticles binding was performed as described [14]. In brief 10 pmole of probe was added to a mixture of 15 μL of gold nanocolloid (120 ppm). After mixing, 1 μL of 10-fold dilution of LAMP products were added to the reaction, heated at 65 °C 5 min and allowed the reaction to be hybridized at room temperature. Signal detection was by adding 1 μL of 100 mM PBS pH 7.0 solutions to the reaction. Changing of gold nanocolloid was observed with the naked eye and measured through UV-Visible Spectroscopy (Ocean Optics^®^ USB2000, Dunedin, FL, USA) with λ = 400–800 nm.

### 2.5. Reaction Validation

Specificity and sensitivity of the LAMP primer set were established as described [28,29]. Primer specificity was determined using DNA from the described target (*Ehrlichia canis*) and non-target bacteria (*Ehrlichia chaffeensis*, *Anaplasma phagocytophila*, *Babesia canis*, *Hepatozoon canis*, and *Escherichia coli*) as the templates. For primer sensitivity, the *p30* element was amplified via PCR using F3 and B3 primer with Go Taq^®^ Green Master Mix (Promega, Madison, WI, USA) as described in manufactured protocol with condition of; 40 rounds of PCR with denaturation at 93 °C 45 s, annealing at 53 °C 60 s, and extension at 73 °C 120 s The obtaining *p30* products were cloned into pSCA plasmid using StrataClone PCR Cloning Kits™ (Stratagene, La Jolla, CA, USA), selected and confirmed for the corresponding *p30* sequence using 1st BASE DNA Sequencing Services (1st BASE, Singapore). The detection limit of the reaction was determined using equivalent copy numbers of cloned *p30* gene at 10-fold dilutions as described [29]. All specificity and sensitivity of the assay was also confirmed and compared with that of PCR using either same lot of positive and negative DNAs and the 10-fold dilutions as the template with 25 μL reaction as described [8].

For method validation [30], blind DNA specimens, (N = 80 with 40 specimens positive and 40 specimens negative), with duplication were prepared from pool clinical specimens. They were then investigated for a comparative assay result between LAMP/gold nanoparticles and that of standard PCR. The test was carried out in duplication with 25 μL reactions both for PCR [8] and for LAMP. PCR results were used as standard. Positive result was determined from positive in both replications, and negative result was determined from both negative results in both replications. Result from specimen having different between each replication was judge from the third DNA draw assay using a majority rule (two third rule). False positive was determined from positive LAMP from PCR negative result and false negative result was determined from negative LAMP from PCR positive. Sensitivity and specificity of the LAMP with colorimetric AuNPs detection method were determined based on receiver operating characteristic (ROC) analysis as described by Fawcett [30].

## 3. Results and Discussion

In tropical areas, canine monocytic ehrlichiosis (CME) is still a major dog disease [31]. Diagnosis of this disease based on symptoms alone or in combination with a laboratory assay is not efficient enough for good treatment and control implementation of this disease, especially when the demand occurs in remote areas where the lack of facilities and expertise are the problems. Therefore developing a rapid assay with a simple platform making it possible to perform the assay even on-site where animal health facilities are restricted is very demanding.

Several platforms including biochemical, or serologic assays had been employed in diagnosis of *E. canis* in the past [4,5]. These depended on protein-based tests either through detection of some level of a target protein or a binding of an immunoprotein with enzymatic activity conjugated directly to a target protein. Although some of them could further be developed as easy to use flow strip detection, their protein-protein binding specificity and the sensitivity limitation at the nano-level does not make them effective enough to apply them for an on-site clinical assay. Recently a molecular assay based on targeting specific DNA molecules has been well accepted worldwide. The technique, namely PCR and the recent advanced real time PCR have been employed for disease diagnosis [7–12].

Their common principles rely on multiple steps of temperature controlled incubation operated by a thermocycler device. These allow the DNA replication processes *i.e.*, denaturation for template preparation, primer annealing and DNA extension via DNA polymerization, to be operated repeatedly. Test results are obtained either via 1 h electrophoresis separation of the DNA products in classical PCR or via a costly detection of fluorescence signals used in Real-Time PCR systems. Use of these PCR-based assays, however, depends not only on the device, but also needs technical expertise. Both conditions limit the use of PCR as an on-site assay. Thus, to develop a rapid nucleic acid diagnostic assay, answering the above demand, an alternative platform not only for specific nucleic acid amplification but also rapid nucleic signal detection is required.

In this study *E. canis* detection was based on a combination of the isothermal DNA amplification technique namely Loop-mediated isothermal amplification (LAMP) and a colorimetric assay using nucleic acid hybridization coupling target recognition with nanoparticle (NPs) aggregation.

**Figure 2.**
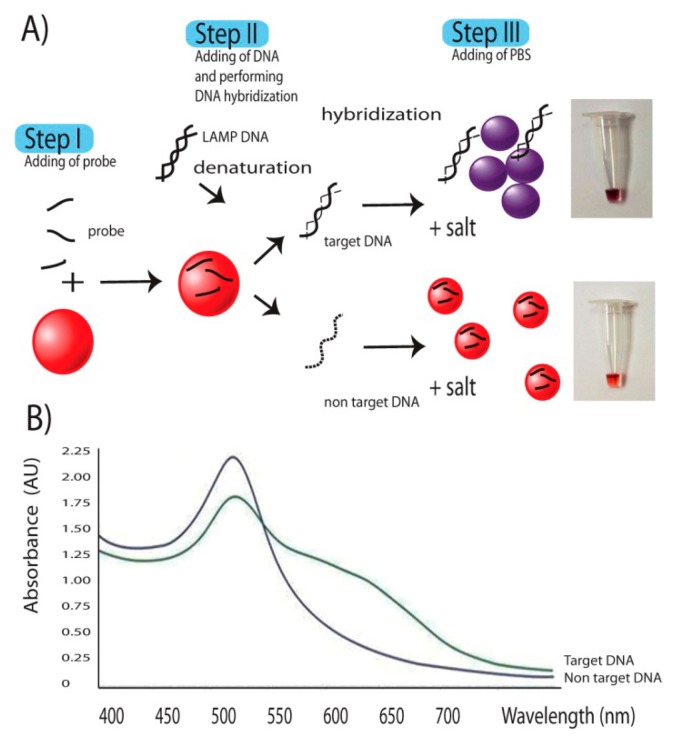
Schematic of a colorimetric DNA detection of *p30* DNA based on gold nanoparticles and its corresponding change in UV-Vis spectrum.

LAMP had been demonstrated since 2000 [13]. The technique relies on a strand displacement DNA synthesis by the large fragment of *Bst* DNA polymerase in a self-cycle style, making the amplification possible at a single temperature without using a thermocycler. It is different from PCR in that the amplification is based on highly specific 4–6 primer initiations making it more reaction specific than that of PCR [32,33]. Compared to PCR and Real-Time PCR, LAMP also has the advantages of reaction simplicity and higher amplification efficiency [13], allowing one to obtain better yield of DNA products (10^9^ copies) in less than an hour. The final products are stem-loop double strand DNAs with several inverted repeats of the target DNA. Further step of DNA signals detection was as described in the Figure 2. The key to the successful signal detection here was the control of particle dispersion and aggregation with target DNA products of interest and oligonucleotide probe. Addition of single strand oligonucleotide probes to AgNPs, as the first step allowed the binding of the probes on the surface of AuNPs via nitrogenous base/gold van der Waals forces [34]. In an *E. canis* positive sample, the target gene was amplified by LAMP resulting in a large amount of several inverted repeats of target double stranded DNA. In a second step the diluted LAMP products were added and denaturation performed by heat incubation at 65 °C for 5 min, opening up a lot of target binding domains which increased the affinity and consumed a large amount of probes. This situation did not happen in *E. canis* negative samples whose DNA amplification and related products were not delivered. During DNA hybridization, a shorter oligonucleotide had tendency to bind to target DNA before the longer DNA counterparts [34]. Hybridization between probes on target domains attracted the majority of probes previously bound on the nanoparticle surface, leading to the change from resistance to salt-induced aggregation to an intolerable one. Then the last step of adding 1 μL of 100 mM PBS pH 7.0 solutions to the reaction induced particle aggregation which in turn provided a colorimetric change of the AuNP colloid from ruby red to purple. This colorimetric change can be measured via its absorbance spectrum on a UV-vis photospectrometer. The aggregated particles exhibited a plasmon shift from 550 nm to 600–610 nm as seen in Figure 2B while no such a change was observed in non-aggregated ones.

**Figure 3.**
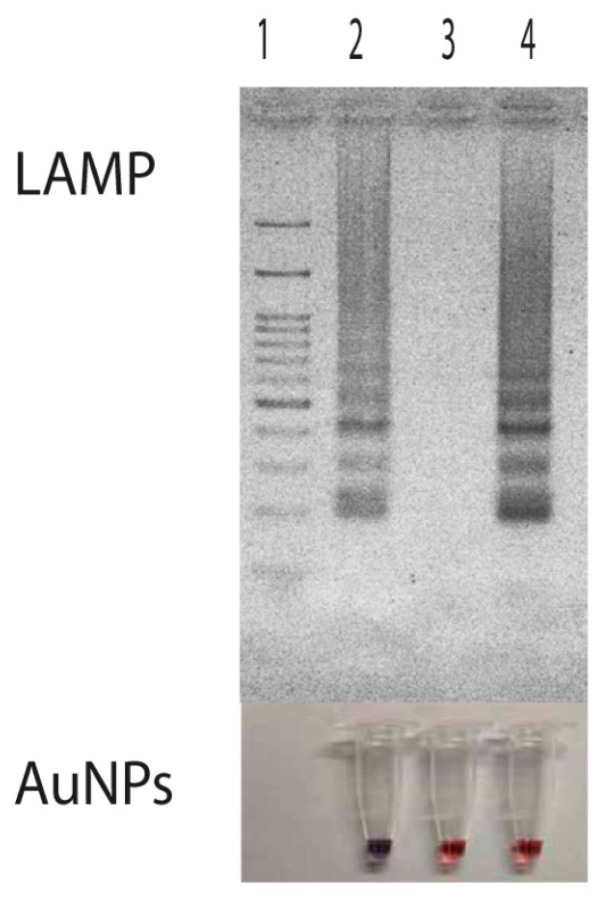
Amplification of *p30* gene by LAMP and its signal detection based on gold nanoparticles. Lane 1, 100 bp marker; Lane 2, *Ehrlichiacanis* DNA; Lane 3, non template control. *p30* gene and Lane 4 non target *Listeria monocytogenase* DNA from LAMP products.

**Figure 4.**
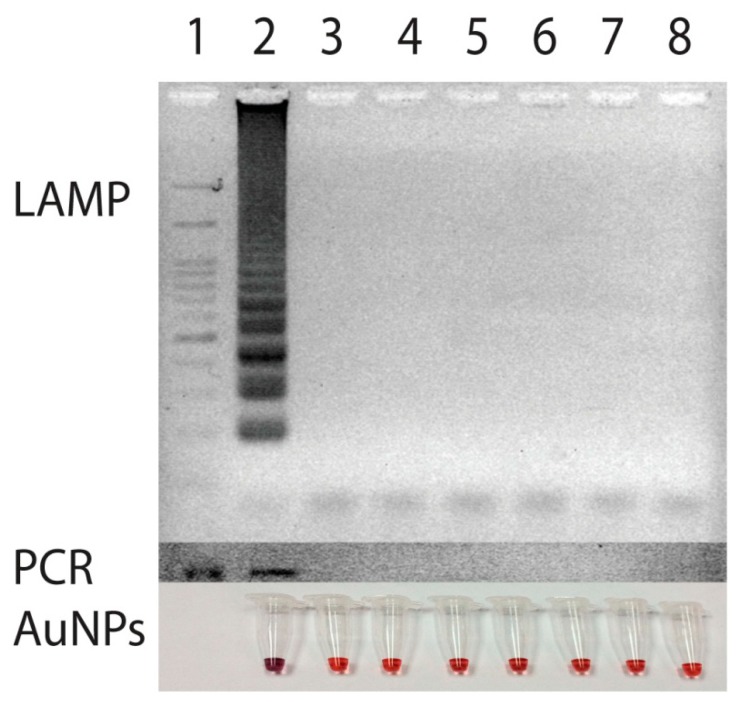
Specificities of LAMP amplification compared with that of PCR and gold nanoparticles detection, Lane 1, 100 bp marker. Amplification and detection were also carried out using DNA from Lane 2, *Ehrlichia canis*, Lane 3–7, negative control, *Ehrlichia chaffeensis*, *Anaplasmapha gocytophila*, *Babesia canis*, *Hepatozoon canis*, and *Escherichia coli*, respectively, Lane 8, non-template control. The amplification and detection of target gene could be obtained only from the template of *E. canis*.

Thus, as a result for an *E. canis* positive sample, AuNPs changed the color from ruby red to purple due to particle aggregation. No such a color change was observed in the result from *E. canis* negative samples (Figures 3 and 4) where the involved particles remained in protected status and dispersed thoughout the tube.

In LAMP system development, selection of the target gene for primer design is an important initial step. For *E. canis* assay, a *16S*rRNA gene had been reviewed and used as target gene in several PCR assay models due to its commonality but specificity to bacterial species [7,10,35]. Detailed inspection of these models revealed a technical limitation in *16S*rRNA gene usage for LAMP development because all used models required a nested PCR platform for their DNA amplification. These reflect the limit in amplification efficiency. Moreover a sequence comparison study, based on *16S*rRNA among strains that cause ehrlichiosis-like symptoms (*Hepatozoon canis*, *Babesia canis*, *Ehrlichiosis chaffeensis*, and *Anaplasma phagocytophila*), showed similarity, especially among the sequences of *E. canis*, *E. chaffeensis* and *Anaplasma phagocytophila*. These made primer design using *16S*rRNA as a target gene candidate difficult.

In response to this, our LAMP system for *E. canis* was focused on the amplification of target *p30* gene. The use of *p30* as a target instead of other genes was because the gene was found only in *E. canis* and *E. chaffeensis* [36]. Previous detection of *p30* by PCR provided more efficiency because there were more copies of *p30* per genome than that of *16S*rRNA [8]. The *p30* gene is also an immune-determinant major outer membrane protein gene of *E. canis* commonly used in immunodetection [36,37]. Moreover, based on sequence variations, our designed primer set was specific only to *E. canis* but not the rest. This contrasted with the result from the recent LAMP detection system of a heat shock gene *groESL* of Faggion, *et al.* [38], whose specificities were crossed with more pathogen species.

Then, a primer set specific to target *p30* gene (from nucleotide position 21399 to 21604 with repect to the sequence domain of Accession No. AF078553) was used (Table 1 and Figure 1). It consisted of F3 (5′-AGTGGAAAGTATGTACCAAGT-3′) and B3 (5′-TAACCGATAGCTCCTGCA-3′) (for the forward and backward primer, which are specific to domain 21399 to 21419 and 21587 to 21604, FIP (forward inner primer) which was composed of F1c (5′-TCCATCCCAATCATGTTTTAATCCA-3′) and F2 (5′-GTCTCACATTT TGGTAGCTTCT-3′) specific to domains 21479 to 21503 and 21420 to 21441, and extra TTT nucleotide between the junction, BIP (backward inner primer) which is composed of B1c (5′-TAAACACGCTGACTTTACTGTTCC-3′) and B2 (5′-CCCTAGAAATGGAT TGTTCTC-3′) specific to domains 21521 to 21544 and 21564 to 21584 and extra TTT nucleotide between the junction, covering all six areas of the *p30* gene. The extra TTT was used here to facilitate the amplification stability of LAMP [13]. When this primer set was used to amplify genomic DNA of *E. canis* at 63 °C for 40 min, it provided a typical ladder pattern of DNA products with a multiplied size of 206 nucleotides. Amplification by LAMP was successful only when *E. canis* positive DNA was in the sample. There were no cross-reaction and non-specific cross of the primer set itself to the target DNA as judged from the clear specific band in the positive lane and the absence of any non-specific band in the negative lane (Figure 3). The LAMP result also agreed with that of PCR [8].

To test for specificity of the primer set, an attempt to amplify DNA from various strains of bacteria including *Ehrlichia canis*, *Ehrlichia chaffeensis*, *Anaplasma phagocytophila*, *Babesia canis*, *Hepatozoon canis*, which cause common symptoms of high fever with depression in canine species were made and compared with that of PCR [8]. For negative control, DNA from *Escherichia coli* was also employed. The amplification of target gene could be obtained only from the template of *E. canis* (Figure 4). Neither nonspecific amplification of DNA products nor a primer dimer was observed when the other DNA was used as the template. Thus, the amplification of *p30* gene primers via LAMP in this study undoubtedly took place only when *E. canis* was present.

**Figure 5.**
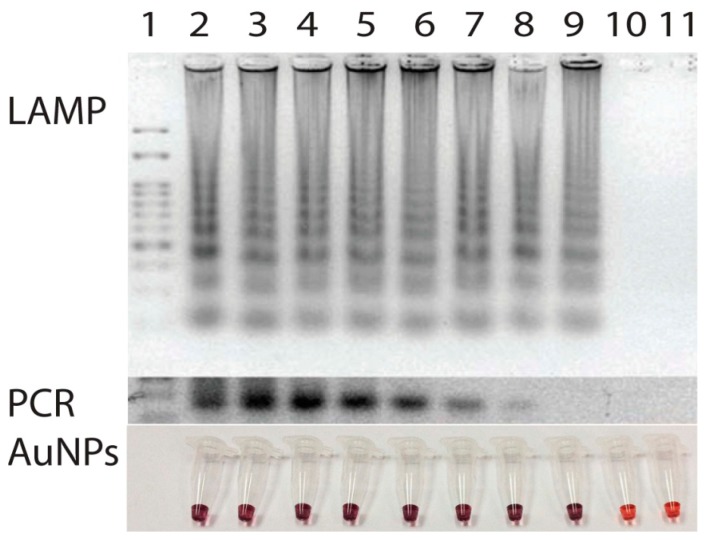
Sensitivities of LAMP amplification and gold nanoparticles detection. Lane 1, 100 bp ladder; Lane 2, Positive control; Lane 3–8, serial dilution of *E. canis* DNA template 10^8^ to 10^2^ copies; Lane 9 and 10, serial dilution of *E. canis* DNA template at 50 and 0 copy; Lane 11, Non template control. LAMP amplification had a limit of detection at 50 copies of *p30* gene.

Similarly, the sensitivity of the primer was tested using the serial dilutions of target *p30* gene in pSCA plasmid form (Stratagene, La Jolla, CA, USA) at different concentrations ranging from 0 to 10^8^ copies. Once DNA amplifications were performed, the results revealed a limit of detection at 50 copies of the *p30* template (Figure 5). Use of LAMP here not only enabled target *p30* gene to be amplified in a short period of time but also the isothermal temperature used opened an opportunity to perform the test on-site with less reliance on laboratory facilities. Based on our experience, with only a simple incubator or polyurethane box that can maintain 63 °C for 40 min, one can easily perform the LAMP reaction in the field.

For DNA signal detection, a colorimetric assay using non-functionalized AuNPs and oligonucleotide probe was developed. In the past, Li and Rothberg [34] were the first who pioneered the use of non-functionalized AuNPs for PCR detection. They took advantage of electrostatic differences between single strand DNA (ssDNA from left primers in PCR negative) and double strand DNA (dsDNA, PCR positive products). Repulsion between the charged phosphate backbone and the adsorbed ions on AuNPs dominates the electrostatic interaction between the particles and dsDNA so that dsDNA will not adsorb while ssDNA is flexible enough to partially uncoil its bases, so they can be exposed to the surface of nanoparticles. This allows the attractive van der Waals forces between the bases and the nanoparticles to interact and cause ssDNA to stick to the particles. Thus at low concentrations of salt, adsorption of ssDNA stabilizes the nanoparticles against aggregation. The same mechanism does not occur with dsDNA because its structure does not permit the bases to be exposed and no such stabilization take place. In this case, AuNPs start aggregation. However if there was, by chance, a non-specific amplification or false positive DNA product, this assay would not distinguish any differences among those false results. To correct this gap, nucleic acid hybridization with probe was a needed solution. Peptide nucleic acid was selected as a probe of choice in the AuNP assay by Kanjanawarut and Su [19] because of its properties of both thermal stability and independence on salt concentration for hybridization, enabling hybridization to occur at room temperature. However, application of this would be wider if there were no technical limitation during PNA synthesis [20,21], and PNA were more available and affordable on the market.

In this study, standard oligonucleotide but not PNA was instead used as probe for nucleic acid hybridization because oligonucleotides are common, cheap and have less limitations when performing a custom synthesis. The probe was selected and designed based on several criteria: (1) its nucleotide sequence should be located between F3 and B3 without interfering with any primer sequences and (2) its length was determined based on Tm that needed to be set close to that of the LAMP primer. Here an oligo of 31 nucleotides in length was chosen. Binding of probe to target DNA was at the site between the F2 and F1 primer (position 21,444 to 21,474) without any hindrance with any primer sequences in the system. The key to the detection platform here was the control of particle dispersion and aggregation with the target *p30* DNA products of interest and the oligonucleotide probe as previously described in Figure 1. Adding a single strand oligonucleotide probe targeting *p30* gene to AuNPs allowed the binding of the probes on the surface of the nanoparticles. The presence of several inverted repeats of target double stranded *p30* DNA products of LAMP positive guarantee the amount of the probe target elements in the system. LAMP had been reviewed, and in terms of productivity, it produces more amount of DNA products (3–8 orders) than PCR [14,39]. Addition of the diluted LAMP products and denaturation then opened up a lot of target binding domains which attracted the affinity of probes. During DNA hybridization, shorter oligonucleotides have a tendency to bind to target DNA before the longer DNA counterparts [29]. Hybridization between probes and *p30* domains consumes a large amount of probes on the nanoparticle surface. This left for the remaining probe binding on the particles less amount than the level required to protect particles from salt-induced aggregation in a later step. Thus, in the *E. canis* positive sample, AuNPs changed color from ruby red to purple due to particle aggregation (Figure 2). In contrast, there was no DNA product from the LAMP reaction from *E. canis* negative samples which in turn failed to initiate such a nucleic hybridization. This left all remaining probe still intact on the surface of the particles, leading to the resistance to salt-induced aggregation. Thus in *E. canis* negative samples, there was no change of the ruby red color of the AuNPs (Figure 2).

Persistent unchanged color of AuNPs was also observed in samples having non-target DNA. Here, we demonstrated the use of LAMP products from *Listeria monocytogenes hly* gene as a non-target DNA for the attempt of nucleic acid hybridization due to its availability in our laboratory. Results showed that the presence of the *hly* domain in LAMP products did not induce any DNA hybridization with the probe used. Since the hybridization did not take place, remaining probes still attached with particles thus protecting them from the salt-induced aggregation (Figure 3, Lane 4). The demonstration of this non-target DNA in correlation with non-aggregation of particles indicated that the aggregation of particles was directly involved with nucleic acid hybridization rather than the presence of double stranded DNA as found in Li and Rothberg's study [34].

The assay system using LAMP and AuNPs here is an example of how to customize LAMP and probe for selective targets to make a colorimetric assay in terms of a qualitative test. In the future, further development of the detection platform for quantitative study will become necessary. So far no such a quantitative study on AuNPs was accomplished yet. However, since the productivity of LAMP during DNA amplification is increased with time in an exponential manner [14], the challenge to develop a semi-quantitative test based on the rate of AuNP aggregation may be possible in the near future.

The colorimetric change was strongly confirmed by the UV-Vis spectrum (Figure 2). The aggregated particles exhibited a plasmon shift with peak appeared from 550 nm to 600–610 nm, depending on time and degree of aggregation. Measurement by UV-Vis spectrophotometry provides an alternate choice for result determination when a concrete decision based on digital data is needed. This spectrum measurement also help any person who had a problem with red color blindness to discriminate the gold signals (from ruby red to blue).

Salt-induced aggregation was also clearly observed in samples having low amount of *E. canis p30* gene near the limit of detection. This was due to the performance of the LAMP technique in delivering more amount of DNA than that of PCR [12,13]. When this was combined with AuNP-based detection, reliable results could then be guaranteed (Figure 5).

Detection of *E. canis p30* gene using DNA from field specimens (N = 80) was also carried out. All specimens used to prepare blind samples were already checked for the presence and absence of *E. canis*. Results from PCR with gel electrophoresis were used as standard. It was found that when the DNAs were tested by both PCR and LAMP, results from each duplicate set were all in agreement. No such pair needed the third sample draw test. All comparative results were summarized in Table 2. LAMP showed one specimen false positive in both replications and one specimen false negative in both replications when compared with the PCR data. One possible reason for the false positive was a carryover contamination during sample preparation. LAMP is sensitive to small amounts of template. It is recommend to carry out LAMP separately from a PCR environment [14] as the presence of the small amount of DNA via a chance carryover might interfere with LAMP amplification. One reason for the other false negative was the DNA target. Since LAMP operates with more primers and all need to be hybridized accurately, nucleotide by nucleotide, in order to initiate the strand displacement DNA amplification [13]. This might not exclude the possibility of mutation in the sequence template involved making it bind incompletely to the primers. Therefore the sequence of this specimen was investigated. For method validation, receiver operating characteristic (ROC) analysis was used for the comparison between LAMP/AuNPs and that of PCR. Based on the results, LAMP with plasmonic change of AuNPs provided 39 of 40 positives, and 39 of 40 negatives compared with PCR with gel electrophoresis [8] as a standard. Specificity calculated from Specificity = true negative/(false positive + true negative) × 100 was 97.50% and sensitivity calculated from Sensitivity = true positive/(true positive + false negative) was 97.50%. Both indicated an acceptable liability to use this method for on- site *E. canis* screening assay.

By the combination of LAMP and DNA signal detection via plasmonic change of AuNPs, a simple assay applicable to onsite monitoring of *E. canis* could be made possible. This provided several advantages both in terms of test speed, with satisfactory results and the ability to perform the test with less dependence on laboratory facilities. Results could be checked simply by the naked eye. Though, this is a qualitative assay, it can be as cheap as approximately 6 US$ per test (3 US$ for chemicals in the detection system and 3 US$ for the DNA extraction kit) and took less than 90 min (40 min DNA extraction, 50 min LAMP with AuNPs detection) for the overall processes.

## 4. Conclusions/Outlook

A simple, rapid, high efficiency and inexpensive assay for detection of *Ehrlichia canis* was developed. This was based on DNA amplification by LAMP and a detection of DNA products by colorimetric change of gold nanoparticles using oligonucleotide probe. It provided 97.50% of specificity and 97.50% of sensitivity with a limit of detection at 50 copies of target *p30* gene. All the steps were complete in 90 min without relying on laboratory facilities. Combining both LAMP and colorimetric detection based on gold nanoparticles enabled us to do the assay rapidly with less dependence on laboratory facilities, with advantages for a point-of-care test.

## Figures and Tables

**Table 1.** Primers and probes used in the assay and its corresponding location to the gene.

Primer	Sequence (5′ to 3′)	Reference Position ^A^
F3	AGTGGAAAGTATGTACCAAGT	21,399–21,419

B3	TAACCGATAGCTCCTGCA	21,587–21,604

FIP (F1c-F2)	TCCATCCCAATCATGTTTTAATCCA*TTT*	21,479–21,503
	GTCTCACATTT TGGTAGCTTCT	21,420–21,441

BIP (B1c-B2)	TAAACACGCTGACTTTACTGTTCC*TTT*	21,521–21,544
	CCCTAG AAATGG ATTGTTCTC	21,564–21,584

PROBE	GCTAAAGAAGAAAGCAAATCAACTGTTGGAG	21,444–21,474

A Sequence *p30* gene, Accession No. AF078553.

**Table 2.** Comparison of PCR with LAMP and AuNPs of *p30* gene from 80 specimens (N = 80).

Specimens	PCR with Gel Electrophoresis ^A^	LAMP with Colorimetric AuNPs Detection
Positive	40	39
Negative	40	39
False positive	-	1
False negative	-	1

Sensitivity ^B^		97.50
Specificity ^B^		97.50

A [8];

B Sensitivity = true positive × 100/(true positive + false negative), Specificity = true negative × 100/ (false positive + true negative).
